# A Metabolomic Study of *Epichloë* Endophytes for Screening Antifungal Metabolites

**DOI:** 10.3390/metabo12010037

**Published:** 2022-01-04

**Authors:** Krishni Fernando, Priyanka Reddy, Kathryn M. Guthridge, German C. Spangenberg, Simone J. Rochfort

**Affiliations:** 1 AgriBio, Centre for AgriBioscience, Agriculture Victoria, Bundoora, VIC 3083, Australia; krishni.fernando@agriculture.vic.gov.au (K.F.); priyanka.reddy@agriculture.vic.gov.au (P.R.); kathryn.guthridge@agriculture.vic.gov.au (K.M.G.); german.spangenberg@agriculture.vic.gov.au (G.C.S.); 2 School of Applied Systems Biology, La Trobe University, Bundoora, VIC 3083, Australia

**Keywords:** pasture and turf protection, known unknowns, bioassay-guided extraction, untargeted analysis, *Epichloë* derived bioprotectives, antifungals, bioassay

## Abstract

*Epichloë* endophytes, fungal endosymbionts of Pooidae grasses, are commonly utilized in forage and turf industries because they produce beneficial metabolites that enhance resistance against environmental stressors such as insect feeding and disease caused by phytopathogen infection. In pastoral agriculture, phytopathogenic diseases impact both pasture quality and animal production. Recently, bioactive endophyte strains have been reported to secrete compounds that significantly inhibit the growth of phytopathogenic fungi in vitro. A screen of previously described *Epichloë*-produced antifeedant and toxic alkaloids determined that the antifungal bioactivity observed is not due to the production of these known metabolites, and so there is a need for methods to identify new bioactive metabolites. The process described here is applicable more generally for the identification of antifungals in new endophytes. This study aims to characterize the fungicidal potential of novel, ‘animal friendly’ *Epichloë* endophyte strains NEA12 and NEA23 that exhibit strong antifungal activity using an in vitro assay. Bioassay-guided fractionation, followed by metabolite analysis, identified 61 metabolites that, either singly or in combination, are responsible for the observed bioactivity. Analysis of the perennial ryegrass-endophyte symbiota confirmed that NEA12 and NEA23 produce the prospective antifungal metabolites in symbiotic association and thus are candidates for compounds that promote disease resistance *in planta*. The “known unknown” suite of antifungal metabolites identified in this study are potential biomarkers for the selection of strains that enhance pasture and turf production through better disease control.

## 1. Introduction

Perennial ryegrass and tall fescue, widely utilized for pasture and turf, are the key source of feed for grazing animals across agricultural industries globally. Pasture yield, quality, and performance directly affect animal health performance, product quality, and industry profitability. Therefore, pasture species and varieties are extensively studied for their yield properties and performance traits to identify the best performing pasture grasses suitable for a vast range of climatic regions throughout the world. Yield, growth parameters, digestibility, nutrient concentrations, abiotic and biotic stress tolerance are some of the characteristics widely studied [1,2,3,4]. For decades genetic research has been carried out to develop pasture cultivars that are better performing in target climates [5,6,7]. 

*Epichloë* sp., of the family Clavicipitaceae, are fungal symbionts of cool-season grasses from the Poaceae sub-family Pooideae [8,9]. *Epichloë* sp. endophytes improve host grass performance by providing the benefits of plant growth promotion, abiotic stress tolerance, pest tolerance, and disease resistance to the host plant by the production of functional secondary metabolites [10,11,12,13,14,15,16]. Due to the importance of these alkaloidal metabolites in animal welfare and pest protection, comprehensive investigations of their biological activity [17,18], mode of action [19,20], and quantitative levels in grasses infected with *Epichloë* sp. endophytes have been reported in the literature [21,22]. The genes and biosynthetic pathways for lolitrem B [23,24,25], epoxy janthitrems [26], ergovaline [27] peramine [28,29], and the lolines [23,30] have also been described and extensively characterized.

Some *Epichloë* sp. endophytes produce alkaloid toxins that are undesirable in an agricultural scenario as they are detrimental to grazing mammals [17,31]. For example, in response to environmental conditions such as high temperature, lolitrem B produced by some *Epichloë festucae* var. *lolii* strains in association with perennial ryegrass can cause ryegrass staggers in cattle and sheep and ergovaline produced by *Epichloë* sp. in association with tall fescue or perennial ryegrass can cause fescue toxicosis to cattle, sheep, and horses [19,32,33]. However, alkaloids such as ergovaline, peramine, and the lolines also provide pest protection to the host plant, which is beneficial in both forage and turf industries as well as in ecological settings [21,34,35].

*Epichloë* sp. endophytes are also capable of improving disease resistance of host grasses, mediated by secondary metabolites possessing antimicrobial activity [18,36,37]. Pasture and turf grasses are threatened by many pathogens that cause devastating and costly losses; some examples include *Puccinia coronata* (crown rust), *Puccinia graminis* (stem end rust), *Pyricularia grisea* (grey leaf spot), *Ceratobasidium cereale* (yellow patch), *Drechslera* sp. (blights and blotches), and *Fusarium* sp. (fusarium patch) [10,38,39,40,41,42,43,44,45]. Thus, improved disease resistance is a desirable trait for both pasture and turf industries. *Epichloë* endophytes that do not produce anti-mammalian toxins have recently been identified as potential candidates for improving host plant disease resistance [45].

Traditional methods of evaluating endophyte-mediated disease resistance involve in vitro detached leaf assays [42,46], *in planta* glasshouse trials [46,47,48], and field trials [49,50,51]. Endophyte-mediated disease resistance has been demonstrated by the use of *in planta* assays and field trials [42,44,47,48,49,50,52,53,54,55]. Tian et al., (2008) described a reduction in lesion size on detached leaves and pot trials of endophyte-infected perennial ryegrass plants when infected with the pathogens *Alternaria alternata*, *Biolaris sorokiniana*, *Curvularia lunata,* and *Fusarium avenaceum* [46]. Li et al., (2018), using pot trials, showed that *Epichloë festucae* var. *lolii* reduced disease incidence in perennial ryegrass plants when infected by *Bipolaris sorokiniana* [48]. However, glasshouse and field trials are particularly complex, time-consuming, and costly to set up. In addition, experimental designs involve varying environmental and ecological conditions that could strongly influence the outcome of the study.

Information regarding strain is not often provided in these studies; however, it is most likely that an *Epichloë festucae* var. *lolii* strain, SE (Standard Endophyte; also referred to as wild type or common endophyte, CE), was investigated rather than the animal-friendly strains utilized in pastoral agriculture. Fernando et al., (2020) showed that the SE strain was indeed bioactive using in vitro bioassays; however, SE also produces toxins detrimental to animal welfare and is therefore not suitable for use in pastures [45,56]. Thus, to better exploit endophyte-mediated disease resistance, improve pasture and turf quality and reduce the impact of phytopathogen disease on animal welfare, animal-friendly *Epichloë* sp. endophyte strains should be investigated. 

Metabolite analysis and identification of antifungal metabolites in vitro and *in planta* allow for better prediction of an *Epichloë* sp. strains performance in disease control before investing in glasshouse and field trials. A previous study by Fernando et al., (2020) [45] showed that two animal-safe *Epichloë* sp. endophyte strains, NEA12 and NEA23, exhibited in vitro antifungal activity against the grass pathogens *Ceratobasidium* sp., *Fusarium* sp., and *Drechslera* sp. The differential bioactivity observed between the two strains examined also indicated variation in the production of bioactive metabolites and their composition. Importantly, the in vitro antifungal phenotypes observed by Fernando et al., (2020) were consistent; observed in independent isolates, across duplicate assays, and under two different culture environment conditions; solid plate-based dual culture assays and agar well diffusion assays using extracts of liquid media supernatant. A second study by Fernando et al., (2021) established that the known antimammalian and insecticidal alkaloids are not responsible for the antifungal activity observed in bioactive *Epichloë* endophyte strains [57]. However, the bioactive compounds should be discoverable using bioassay-guided fractionation and isolation. 

The bioassay-guided isolation of compounds is a common method used in natural products chemistry to characterize bioactive compounds produced by plants or microbes [58]. Plants and microbes tend to produce a vast range of bioactive compounds, from small molecules such as fatty acids and terpenoids to large proteins. Though their chemical properties can be evaluated by many analytical techniques (UV, mass-spectrometry), their biological activity can only be studied using bioassays [59,60]. In bioassay-guided extraction, each step of extraction, fractionation, and purification are tested for the target activity, using a suitable bioassay to identify the extracts/fractions containing the bioactive compounds. This process is important to identify and isolate the compounds responsible for the biological activity.

This study investigates antifungal activity mediated by endophyte-derived metabolites by conducting an untargeted metabolite analysis of two bioactive *Epichloë* sp. endophyte strains, NEA12 and NEA23, first identified in an in vitro dual culture bioassay screen [45]. This approach is led by the bioassay-guided extraction of antifungal metabolites and ‘mining’ in vitro and *in planta* metabolomes for endophyte-derived bioactive candidates. The annotated ‘known unknown’ metabolites from bioactive extracts could be utilized as biomarkers to characterize the antifungal activity of in vitro or *in planta* extracts of *Epichloë* sp. endophytes and thus assist in the selection of superior endophyte × cultivar combinations.

## 2. Results

### 2.1. Extraction of Antifungal Metabolites

**Figure 1:**
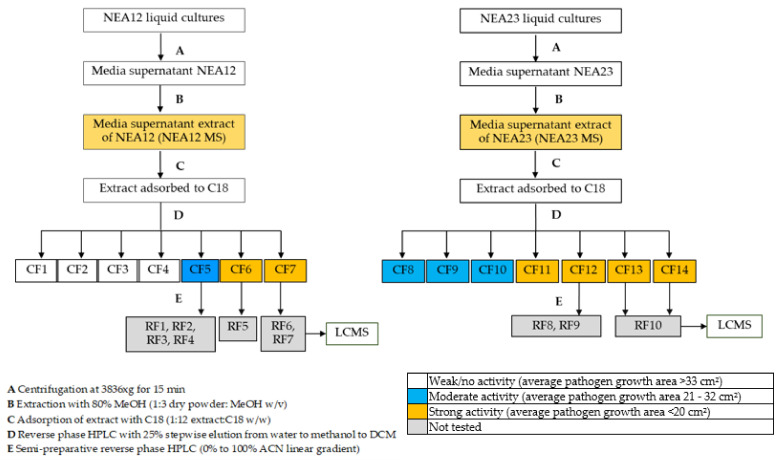
Step-wise bioassay-guided fractionation scheme for obtaining refined fractions of antifungal metabolites.

Media supernatant from *Epichloë* sp. endophyte strains, NEA12 and NEA23, grown in in vitro culture was extracted in 80% methanol yielding two media extracts (NEA12 MS and NEA23 MS). Seven crude fractions (CF) were then obtained from each MS extract as described in the isolation scheme (Figure 1) using high-performance liquid chromatography (HPLC) for separation and liquid chromatography—mass spectrometry (LCMS) for metabolite profile analysis. Subsequently, all MS and CF extracts were subjected to agar well diffusion assays against the phytopathogen *Ceratobasidium* sp. (VPRI 22537) to assess antifungal activity. Bioactivity was visually characterized by three pathogen growth parameters: growth area, mycelial density, and direction of growth. Image analysis using Image-J software allowed quantification of the pathogen growth area.

**Figure 2:**
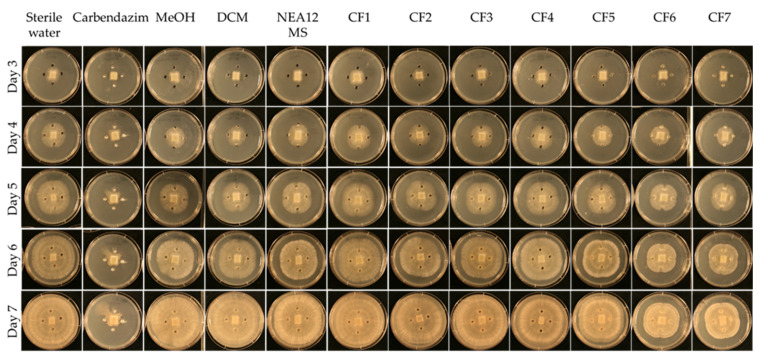
Agar-well diffusion assay for pathogen *Ceratobasidium* sp. in the presence of NEA12 MS derived crude fractions (CF). From left to right: Sterile water; antifungal compound carbendazim (1000 ppm); 80% methanol; 100% DCM; NEA12 MS; CF1; CF2; CF3; CF4; CF5; CF6 and CF7. All bioassay treatments and controls were prepared in replicates of five (*n* = 5). Temporal variation is described in the rows from day three to seven. The images are a typical representation of the five replicates.

**Figure 3:**
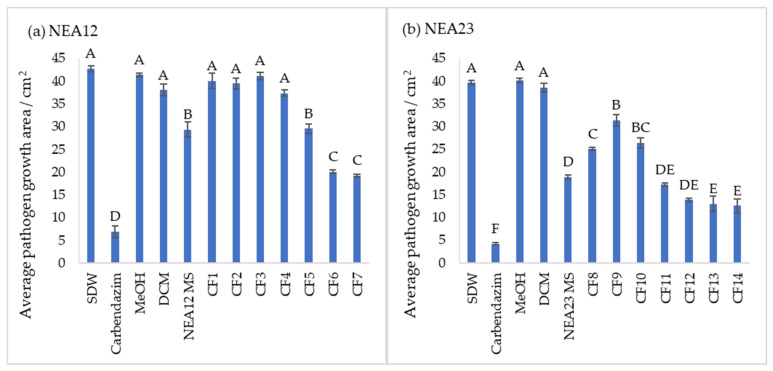
Growth inhibition of *Ceratobasidium* sp. pathogen by *Epichloë* spp. in vitro culture extracts at day 6 (**a**) NEA12 MS and derived crude fractions (CF1, CF2, CF3, CF4, CF5, CF6, CF7); (**b**) NEA23 MS and derived crude fractions (CF8, CF9, CF10, CF11, CF12, CF13, CF14). Controls, sterile distilled water (SDW); carbendazim (1000 ppm); 80% methanol (MeOH), 100% dichloromethane (DCM). Image analysis measured growth area (cm^2^) of the pathogen in the agar well diffusion assay. All data are mean ± standard error, *n* = 5. Significance was determined by one-way ANOVA and Tukey simultaneous tests for differences of means; *p* < 0.01 indicates significant inhibition. Means that do not share a letter are significantly different.

In the assay, the NEA12 MS-derived fractions CF6 and CF7 displayed strong activity against *Ceratobasidium* sp. (pathogen growth area < 20 cm^2^, Figure 2 and Figure 3a). Strong bioactivity was characterized by a smaller growth area and lower mycelial density compared to the negative controls. This was evident throughout the duration of the bioassay. CF5 moderately inhibited the growth of the pathogen compared to the controls (pathogen growth area 21–32 cm^2^). CF1, CF2, CF3, and CF4 didn’t display activity against *Ceratobasidium* sp. 

A one-way ANOVA analysis compared the effect of twelve treatments (degrees of freedom = 11), eight antifungal treatments (CF1–CF7 and NEA12 MS), and four controls on pathogen growth area, a measure of antifungal activity. The ANOVA results showed that at least two treatments are significantly different in antifungal activity at 99% confidence (*p* < 0.001) (Table 1). Tukey’s Post-Hoc test grouped the CFs 1–7, NEA12 MS, and controls to four groups at 99% based on the statistically significant mean difference of the average pathogen growth area (Figure 3a). Tukey simultaneous tests for differences of means for pathogen growth area on day 6 confirmed inhibitory activity of CF5 (*p* < 0.001,), CF6 (*p* < 0.001), and CF7 (*p* < 0.001) compared to the respective solvent controls. CF1 (*p* = 0.998), CF2 (*p* = 0.971), CF3 (*p* = 1.000), and CF4 (*p* = 0.216) did not display significant inhibitory activity compared to the respective negative controls. NEA12 MS showed moderate activity, and the activity was significant (*p* < 0.001) compared to its control. Carbendazim (1000 ppm), a systemic benzimidazole fungicide commonly used in laboratory assays and used in this study as a positive control, significantly inhibited phytopathogen growth (*p* < 0.001) compared to the 80% methanol control. Using these observations as a guide, the bioactive crude fractions, CF5, CF6, and CF7, were selected for further analysis.

**Figure 4:**
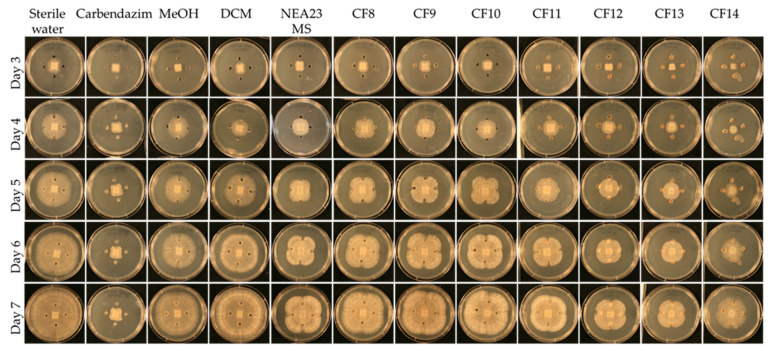
Agar-well diffusion assay for pathogen *Ceratobasidium* sp. in the presence of NEA23 MS derived crude fractions (CF). From left to right: sterile water; antifungal compound carbendazim (1000 ppm); 80% methanol; 100% DCM; NEA23 MS; CF8; CF9; CF10; CF11; CF12; CF13; and CF14. All bioassay treatments and controls were prepared in replicates of five (*n* = 5). Temporal variation is described in the rows from day three to seven. The images are of a typical representation of the five replicates.

In the presence of NEA23 MS and derived crude extracts CF8–CF14, the pathogen was also observed growing away from the agar wells, evident by the ‘X’ like growth pattern. NEA23 MS and fractions CF11, CF12, CF13, and CF14 showed strong activity against the pathogen, as was evident by the smaller growth area and lower mycelial density of the pathogen compared to the negative controls (pathogen growth area < 20 cm^2^; Figure 3b and Figure 4). CF8, CF9, and CF10 displayed moderate activity compared to the negative controls (pathogen growth area 21–32 cm^2^). 

A one-way ANOVA analysis compared the effect of twelve treatments (degree of freedom = 11), eight antifungal treatments (CF8–CF14 and NEA23 MS) and four controls on pathogen growth area which indicates the antifungal activity. The ANOVA results showed that at least two treatments are significantly different in antifungal activity at 99% confidence (*p* < 0.01) (Table 1). Tukey’s Post-Hoc test grouped the CFs 8–14, NEA23 MS and controls to six groups at 99% confidence based on the statistically significant mean difference of average pathogen growth area (Figure 3b). Tukey simultaneous tests for differences of means for pathogen growth area on day 6 confirmed inhibitory activity by CF11 (*p* < 0.001), CF12 (*p* < 0.001), CF13 (*p* < 0.001) and CF14 (*p* < 0.001) and NEA23 MS (*p* < 0.001) compared to the respective solvent controls (Figure 3b). The inhibitory activity of CF8 (*p* < 0.001), CF9 (*p* < 0.001), and CF10 (*p* < 0.001) was also significant compared to the 80% methanol control. The antifungal compound carbendazim displayed strong inhibitory activity (*p* < 0.001) compared to the 80% methanol control. Using these observations as a guide, the highly active crude fractions, CF12, CF13, and CF14 were selected for further analysis.

According to bioassay results, the strongly bioactive crude fractions NEA12 MS (CF6, CF7) and NEA23 MS (CF13, CF14) eluted with non-polar solvent systems (methanol: DCM 1:1 *v*/*v* and 100% DCM). The moderately bioactive crude fractions, having activity similar to that observed by the original MS extracts, eluted with 100% methanol (NEA12—CF5; NEA23—CF11 and CF12), while CFs in water (100%, CF1 and CF8; 80% CF2 and CF9; and 50% CF3 and CF10) exhibited a lower antifungal activity than that observed by the original MS extracts.

### 2.2. Metabolite Profiling

Further fractionation and purification of crude fractions (CF5, CF6, CF7, CF12, CF13, CF14) by HPLC using C18 reverse phase column chromatography provided refined fractions (RF). RF1–RF7 were derived from NEA12 MS CF5, CF6, CF7, and RF8–RF10 were derived from NEA23 MS CF12, CF13, CF14 (Figure 1). 

The LCMS analysis was then used to detect metabolites in refined fractions (NEA12 RF1–7, NEA23 RF8–10) and determine if they are consistently present in both in vitro culture (NEA12 ME and NEA23 ME) and *in planta* (NEA12 PE and NEA23 PE). *In planta* extracts (NEA12 PE, *n* = 48 and NEA23 PE, *n* = 30) were obtained from the pseudo stems of individual genotypes of mature perennial ryegrass-endophyte symbiota. The media supernatant from replicate in vitro cultures (NEA12 ME, *n* = 27 and NEA21 ME, *n* = 28) were also individually extracted and analyzed to determine the reproducibility of the metabolites produced. 

The LCMS spectral data obtained for in vitro (ME), *in planta* (PE), and refined fractions (RF) was analyzed using Refiner and Analyst modules of Genedata Expressionist^®^ to annotate metabolites and compare metabolite profiles. To ensure the metabolites in an in vitro extract metabolome were free of PDB media derived metabolites, sterile media was also extracted, analyzed in parallel by LCMS, and metabolites detected in the media alone were filtered out of the in vitro extract metabolomes. 

**Figure 5:**
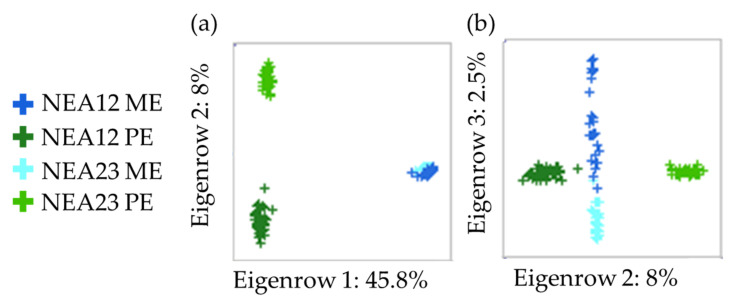
Principal component analysis scores plot (**a**) PC1 and PC2; (**b**) PC2 and PC3 of ESI+ UHPLC-HRMS data of NEA12 *in planta* (NEA12 PE, *n* = 48), NEA12 in vitro (NEA12 ME, *n* = 27), NEA23 *in planta* (NEA23 PE, *n* = 30), NEA23 in vitro and (NEA23 ME, *n* = 28).

As expected, the metabolomes of PE and ME are distinct since the *in planta* and in vitro environments differ, and the former also contains plant-only metabolites that are not found in culture. Principal component analysis (PCA) revealed the separation of PE populations from ME population samples on PC1 (45.8% of the total variation; Figure 5a). Within each population, individual samples are tightly clustered, indicating that the metabolome is reproducible. Importantly, as shown in Figure 5b, there is a small but observable variation between the two different endophyte strains in the same environment—*in planta* and in vitro. The two PE populations (NEA12 PE and NEA23 PE) form distinct clusters on PC2 (8.0% of the total variation), while the two ME populations (NEA12 ME, NEA23 ME) separate out on PC3 (2.5% of the total variation). 

**Figure 6:**
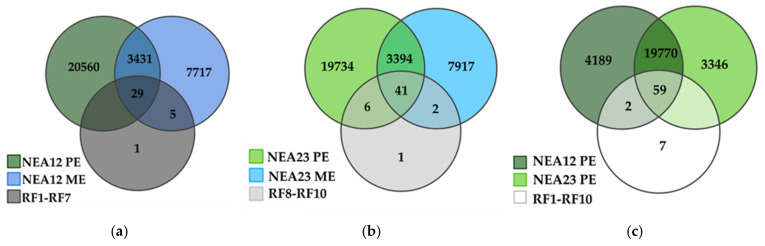
Venn diagram showing the metabolite distribution of (**a**) NEA12 *in planta* (NEA12 PE, *n* = 48), NEA12 in vitro (NEA23 ME, *n* = 27) and NEA12 RF (RF1-RF7); (**b**) NEA23 *in planta* (NEA23 PE, *n* = 30), NEA23 in vitro (NEA23 ME, *n* = 28) and NEA23 RF (RF8-RF10); (**c**) NEA12 *in planta* (NEA12 PE, *n* = 48), NEA23 *in planta* (NEA23 PE, *n* = 30), and RF (RF1-RF10) by ESI+ MS.

Venn diagrams were generated to compare the metabolome of endophytes in different growth environments (in vitro, ME, and *in planta*, PE) and identify the presence of antifungal metabolites in refined fractions (RF) (Figure 6). It is first interesting to note that approximately 30% of the metabolites produced by the endophyte strain in vitro are also present *in planta* (NEA12, 3460/11,182, 30.3%; NEA23, 3435/11,354 30.9%); these metabolites are common to both environments (Figure 6a,b). The presence of an endophyte also impacts the symbiotum metabolome, as shown in Figure 6c, where more than 80% (19,829) of metabolites are in common (NEA12, 19,829/24,020, 82.6%; NEA23, 19,829/23,175, 85.6%), while approximately 16% may be attributed in part due to the presence of different endophyte strains (NEA12, 4191/24,020, 17.4%; NEA23, 3346/23,175, 14.4%). 

Of the metabolites identified in the NEA12 refined fractions (RF1–7), 82% (29/35) are present in both NEA12 ME and NEA12 PE samples (Figure 6a). For NEA23, 82% (41/50) of metabolites identified in the refined fractions (RF8–10) are present in both the NEA23 ME and NEA23 PE samples (Figure 6b). 

The compounds detected in the RF samples that were not detected in both PE and ME (NEA12, 1/35; NEA23, 1/50) are likely impurities introduced from the extraction process. These two compounds eluted very early in the LCMS profile, suggesting they are likely unrelated to the non-polar refined fractions. 

There are also metabolites only detected in in vitro populations (NEA12 ME and NEA12 RF, 5/35; NEA23 ME and NEA23 RF, 2/50). The presence of these compounds likely reflects the different growth environments; in vitro culture compared to the natural environment of the endophyte in symbiotic association. Some metabolites were not observed in the ME samples but were detected *in planta* (NEA23 PE–6 of 50). 

In refined fractions RF1–10, a total of 68 metabolites were detected (Figure 6c). Of these 68 metabolites, 59 were detected in both NEA12 PE and NEA23 PE, two were exclusive to NEA12 PE, and seven were exclusive to the RF fractions. Therefore, the majority of detected prospective antifungal metabolites synthesized in vitro (61/68, 89.7%) are also produced by the endophyte in symbiotic association with perennial ryegrass. 

Of the 68 metabolites detected in RFs, 63 were present in at least one in vitro culture, NEA12 ME and/or NEA23 ME (Supplementary Table S1). The majority of these, 80% (52/63), were observed at a frequency greater than 80% in at least one in vitro secretome (NEA12 ME ≥ 22/27; NEA23 ME ≥ 23/28). From the detected metabolites 14/63 are significantly different (Q < 0.001) in relative abundance between the two in vitro metabolome populations and exhibited greater than 2-fold differential abundance (directed effect size (DES) ≥ 2). The remaining 49 metabolites either did not vary significantly in average relative abundance (Q > 0.001) between populations or exhibited less than 2-fold differential abundance (DES < 2). The DES of significant metabolites was obtained by calculating the ratio of average relative abundances of NEA12 ME: NEA23 ME for each metabolite. 

Table 2 describes the properties of the 61 prospective antifungal metabolites synthesized in vitro, enriched for in refined fractions, and their presence in the NEA12 PE and NEA23 PE symbiota metabolomes. The distribution of these 61 metabolites in refined fractions RF1-RF10 was different from one another (Supplementary Table S2).

Considering the presence of each metabolite, 75% (45/61) were observed in the plant metabolome at a frequency of greater than 80% in at least one *in planta* population (NEA12 PE ≥ 38/48 symbiota; NEA23 PE ≥ 24/30 symbiota). Only 5/61 were observed at a frequency of less than 20% in both populations (NEA12 PE ≤ 9/48 and NEA23 PE ≤ 6/30). The remaining 16% (10/61) were observed more frequently in one symbiotum metabolome, most often NEA12 PE (9/10 metabolites). 

The differences in the relative abundance of metabolites between the two PE metabolomes are described using a Q-value; if the Q-value < 0.01, the relative abundances are significantly different. Variation was further described by looking at the DES of metabolites that were significantly different among the two populations.

Among the 45 metabolites present in at least one symbiotum metabolome population at a frequency greater than 80%, 31 are significantly different (Q < 0.01) in relative abundance. Here, 7/31 are more abundant (DES > 2) in the NEA12 PE metabolome, and 6/31 are more abundant (DES > 2) in the NEA23 PE metabolome. 

Of the metabolites observed more frequently in the NEA12 PE plant metabolome, 4 metabolites are found almost exclusively in the NEA12 PE metabolome (M8, M13, M32, and M43) and a statistical analysis was not possible; 5 metabolites are not significantly different in terms of relative abundance between *in planta* populations; and one metabolite (M29) is significantly different (Q < 0.001) in relative abundance and the DES indicates M29 (11.6) is more abundant in NEA23 PE. The one metabolite observed more frequently in the NEA23 PE metabolome (M21) is more abundant in the NEA12 PE metabolome (Q = 0.024, DES 1:2.386). 

A total of 13/61 metabolites were significantly different (Q < 0.01) in relative abundance between the two symbiota metabolome populations and exhibited a greater than 2-fold differential abundance (DES): M5 (1:3.927), M18 (1:7.060), M19 (2.49), M21 (1:2.386), M23 (3.93), M24 (5.117), M25 (1:2.198), M29 (11.857), M34 (1:4.525), M39 (1:9.063), M42 (1:3.271), M48 (2.038) and M52 (2.477). The remaining 48 metabolites either did not vary significantly in average relative abundance (Q > 0.01) between two symbiota populations or exhibited a less than 2-fold differential abundance (DES < 2). 

Considering the abundance of the metabolites described in Table 2, in most instances the in vitro (RF) samples are enriched for potential bioactive metabolites when compared to the average abundance of the corresponding metabolite *in planta* (PE) (Supplementary Figure S1). Crude extraction of the media supernatant followed by bioassay-guided extraction, from a starting point of mycelia cultured in 10 L of media supernatant, has resulted in refined extracts that are enriched for a suite of metabolites that are candidates for in vitro bioactivity. The presence of these 61 metabolites *in planta* (PE) and refined fractions (RF) further confirms that perennial ryegrass-endophyte symbiota produce these metabolites (Supplementary Figure S1). 

Together this data describe a suite of potentially bioactive ‘known unknown’ *Epichloë* sp.-derived metabolites identified using bioassay-guided extraction and establish that most of the metabolites in the in vitro *Epichloë* sp. secretome are also produced in perennial ryegrass-NEA12 and perennial ryegrass-NEA23 symbiota. 

## 3. Discussion

*Epichloë* sp. endophytes associated with pasture and turf grasses improve the disease resistance of their host grasses. Endophyte-mediated disease resistance is an outcome of complex host-pathogen-endophyte interactions resulting in antimicrobial compound production, secretion, and distribution throughout the host plant [61]. Though this process is not well understood, some studies have identified antifungal metabolites from sexual (pathogenic) *Epichloë* species [18,36,62,63,64,65], in particular *Epichloë* *festucae* [10,44,66], and characterized their effects on phytopathogens. Most studies confirm the antifungal activity using in vitro assays of the endophyte culture itself, culture filtrates, or isolated compounds [10,18,36,45,46,66,67]. In contrast, the investigation into the compounds responsible for endophyte-mediated disease resistance in asexual, vertically transmitted, *Epichloë* species that are utilized in perennial ryegrass and tall fescue are limited. 

In Fernando et al., (2020) [45], an in vitro dual culture screen of asexual *Epichloë* sp. endophyte strains from perennial ryegrass and tall fescue identified bioactive strains that suppressed the growth of phytopathogens. A further investigation of four strains—SE, NEA12, NEA21, and NEA23—revealed the differential bioactivity of the strains against grass pathogens *Ceratobasidium* sp., *Drechsler* sp., and *Fusarium* sp. and identified the endophyte secretome as having higher bioactivity, compared to the mycelia, in liquid culture. A second study reported that the ‘known knowns’—*Epichloë* sp. related antimammalian and insecticidal alkaloids—are not responsible for the observed antifungal bioactivity [57]. Here, pure forms of endophyte-related alkaloids—namely peramine, *n*-acetylloline, *n*-formylloline, lolitrem B, janthitrem A, paxilline, terpendole E, terpendole C, ergovaline, ergotamine, ergocornine, ergocryptine, ergotaminine—were tested against *Ceratobasidium* sp., *Drechslera* sp. and *Fusarium* sp. in agar well diffusion assays [57]. Thus, the opportunity now arises to investigate novel *Epichloë* sp. for their potential in endophyte-mediated disease resistance and identify metabolite biomarkers for bioactive animal-friendly strains. 

For the application and a better understanding of endophyte-mediated disease resistance, studying antifungal metabolite presence and abundance *in planta* is important. In this study, antifungal metabolites responsible, either singly or in combination, for in vitro bioactivity were identified using bioassay-guided extraction of the *Epichloë* sp. endophyte secretome, and their presence and abundance in *Epichloë* sp.-perennial grass symbiota was explored. The secretome—media supernatant (MS) extracts—of two bioactive endophyte strains, NEA12 and NEA23, were fractionated using solvent systems with different polarities to obtain seven crude fractions (CF) from each endophyte strain. These MS extracts and the derived CFs were then tested against the pathogen *Ceratobasidium* sp. in agar well diffusion assays to determine their in vitro antifungal activity. Agar well diffusion assays can be easily replicated and generate accurately quantifiable results.

The NEA12 secretome showed moderate activity, and NEA23 showed strong activity. These observations were consistent with previous findings [45]. NEA12 MS extracts in agar-well diffusion assays exhibited moderate inhibition of *Ceratobasidium* sp.; while the ‘X’ like growth pattern of *Ceratobasidium* sp. was observed in the presence of NEA23 MS, indicating significant growth inhibition [45]. The differential antifungal activity of the two *Epichloë* sp. strains against the same pathogen may be due to differences in the presence and/or abundance of antifungal metabolite in each secretome. It is noteworthy that the two studies were four months apart, an indication that the antifungal metabolites produced by these endophyte strains are stable at −80 °C and at room temperature (22 °C) for the duration of the assay (7 d).

Crude fractions in 1:1 methanol: DCM and 100% DCM (CF6, CF7, CF13, and CF14) exhibited significantly stronger antifungal activity compared to the original MS extracts and other CFs, thus confirming enrichment of antifungal metabolites from the endophyte secretome in these fractions. Thus, the antifungal metabolites are most likely not highly polar compounds as they have eluted with organic solvent systems. 

A Metabolome analysis may entail either a targeted analysis of a certain class of metabolite, or a total metabolite profiling of a given sample, or population of samples. Preliminary screening of the total metabolome provides a view on overall metabolite production; whereas, targeted bioassay-guided isolation and characterization provides a more concise detail about metabolites and their potential uses as antifungal compounds [60]. 

Bioassay-guided extraction/isolation processes are often used to isolate bioactive metabolites from plants and microorganisms [10,58,68,69,70]. Yue et al. isolated six antifungal metabolites, indole-3-acetic acid, indole-3-ethanol, methylindole-3-carboxylate, indole-3-carboxaldehyde, diacetamide, and cyclonerodiol from *Epichloë* sp., in in vitro cultures using a bioassay-guided extraction process which inhibited the growth of *Cryphonectria parasitica*, *Lactisaria fusiformis*, *Magnaporthe poae* and *Rhizoctonia solani* [10].

Strong bioactive crude fractions (CF5–7 and CF11–14) were selected for further characterization. With further fractionation, refined fractions were obtained from both endophyte strains. Complex crude fractions were subjected to another round of chromatography to obtain cleaner refined fractions, which facilitates better identification in terms of abundance and intensity. While further purification is necessary to fully characterize the antifungal metabolites in these refined fractions, LCMS analysis can accurately annotate the metabolites, providing level 5 metabolite identification based on their unique mass, *m*/*z* and retention time (RT) [71,72]. To enhance the accuracy of the refining process, low tolerance thresholds were used (RT alignment ± 0.1 min, isotope clustering RT ± 0.02 min, *m*/*z* ± 0.05 Dalton). Thus, information on chemical properties and elution times can be used to detect the presence of the same metabolites in any matrix, using suitable extraction, and LCMS analysis methods. The exact methods adopted will depend on the chemical characteristics of the active metabolites e.g., polarity, and in some instances, more efficient purifications could be developed e.g., extraction of aqueous fractions using ethyl acetate or dicholormethane. 

Annotated metabolite data of refined fractions, secretome extracts of in vitro cultures (ME), and extracts from populations of endophyte-perennial ryegrass symbiota (PE) of NEA12 and NEA23 were used to determine the presence of metabolites from the refined fractions in replicated samples of the endophyte secretome in vitro and *in planta*.

Tight clustering patterns were observed and show the homogenous nature of the metabolome within in vitro and *in planta* populations. The tight clustering patterns in PCA plots clearly distinguish the in vitro and *in planta* populations, as expected the two growth environments are different. Importantly, a small yet observable separation of the two endophyte strains was noted by the separate clustering of individuals from each population within the two growth environments. This separation represents the difference in metabolomes due to the genetic differences between the two endophyte strains, NEA12 and NEA23. 

Most of the metabolites in refined fractions derived from the NEA12 and NEA23 secretomes are also present in the in vitro and *in planta* populations examined, providing strong evidence for their constitutive and reproducible production. A small portion of RF metabolites (NEA12; 5/35, NEA23; 2/50) were produced only in in vitro culture. These metabolites may be produced exclusively in the in vitro environment or present at trace concentrations *in planta* (and therefore below threshold levels set in this study). Alternatively, they may be due to the introduction of contaminates during the purification process or induced in response to environmental conditions, such as a stress response, that the *Epichloë* sp.-perennial ryegrass symbiota, maintained under optimal conditions in the glasshouse, were not exposed to [73,74,75,76]. 

In this study, about 23,500 features were detected in *Epichloë* sp.-perennial ryegrass symbiota. The two symbiota populations shared about 80% of their metabolome, while about 16% may be attributed to the presence of different endophyte strains. A recent untargeted metabolomic study in endophyte-free perennial ryegrass plants detected about 17,500 features in ESI+ mode after applying filters for accuracy [77]. There is some evidence that *Epichloë* sp. endophyte infection, apart from having different ‘known known’ alkaloid profiles, significantly influences the host plant metabolome [78,79,80]. However, recent studies do not usually distinguish the endophyte metabolome from the plant metabolome in symbiosis [61,81]. By identifying biomarkers from in vitro endophyte extracts, this study has provided a strategy to unravel endophyte-derived bioprotective metabolites from the symbiotia. 

The metabolites conferring antifungal bioactivity in in vitro assays, enriched for using bioassay-guided extraction, and present in perennial ryegrass-endophyte symbiota potentially have a role in conferring disease resistance. Considering the potentially bioactive metabolites detected in refined fractions, the vast majority, 87% (59/68), are also present in both symbiota populations; just two metabolites are exclusive to the NEA12-perennial ryegrass population. This outcome indicates that there is a suite of prospective antifungal metabolites common to the two *Epichloë* sp. strains investigated, and they are also expressed *in planta*.

The two bioactive strains in this study are from different lineages and classified as different species. NEA23, a *Fa*TG-3 (*Festuca arundinacea* taxonomic group-3) isolated from tall fescue [82,83,84] and NEA12 a *Lolium perenne* taxonomic group-3 (*Lp*TG-3) strain isolated from perennial ryegrass [26,85,86]. The two strains exhibit different ‘known known’ alkaloid profiles (NEA12, epoxy janthitrems; NEA23, peramine, *n*-acetylloline and *n*-formylloline) [82,85]. Given the noted genetic and alkaloid profile differences and observed similarity in the metabolite profiles in this study, an in-depth study on the bioprotectives of these two strains can unravel the potential of using biomarkers to identify superior endophyte strains. 

A suite of common potentially bioactive endophyte-derived metabolites that are synthesized *in planta* enables/allows for their utilization as biomarkers for bioprotection. In this study, 75% (45/61) of the metabolites were observed in the plant metabolome at a frequency of greater than 80% in at least one symbiotum population. Metabolites that are exclusive to one symbiota population, as observed for NEA12, may be strain-specific biomarkers and potentially associated with differential bioactivity [45].

Moreover, of interest is the relative abundance of the metabolites *in planta*; only 21% (13/61) of significantly different metabolites vary greater than 2-fold in relative abundance between the two symbiota metabolome populations. The size of the symbiota populations (NEA12 *n =* 48, NEA23 *n =* 30) is large enough to minimize the impact of variation between individual symbiota due to host-endophyte genetic interaction (noting that while the endophytes are clonal, the ryegrass is not) and determine differences if they are present [87,88]. Further investigation is required to determine the effect of the endophyte strain on antifungal metabolite abundance *in planta*. 

Metabolites present in vitro and absent *in planta* may be present in endophyte-perennial ryegrass symbiota at trace levels, below the threshold set for detection in this study. It is also plausible that those metabolites present only in vitro were not identified in symbiota because they are stress-induced [89]. It is possible that the abundance of these in vitro-only metabolites (and endophyte-derived metabolites in symbiota) may change in response to challenge by disease-causing pathogens [89,90]. The environment in which endophyte mycelia is grown in culture, out of its natural environment, and grown in culture for extended periods can activate metabolic pathways and stimulate the production of bioactive compounds; thus, it is likely that some metabolites produced in vitro may not be produced by plants growing in optimal environmental conditions [73,74,75,76]. It would be interesting to further investigate these prospective antifungal metabolites as they could be candidates for bioprotection/abiotic stress tolerance if they are induced by stress. In this study, there is some evidence for the presence of inducible metabolites in the data. A small proportion of metabolites were observed infrequently in symbiota and may be induced in response to environmental stimuli. The refined fractions (RF) may pick up some impurities during the isolation procedure. 

For compound discovery using bioassay-guided isolation, investigating multiple endophyte strains and symbiota populations allows for bioprospecting for novel bioprotective compounds and identification of biomarkers for phytopathogen disease control. The presence of a predictive suite of annotated metabolites, categorized as “known unknowns” [91], in other grass-endophyte symbiota and/or in vitro cultures may provide a way to accurately predict disease resistance conferred by novel *Epichloë* sp. endophyte strains prior to establishing field trials. The robust analysis methods used have allowed the analysis of a larger number of samples in a shorter amount of time and confirmed the consistent presence of detected metabolites in symbiotic associations. This is important as the production of secondary metabolites can be dependent on biotic and abiotic effects. Without consistent production of these metabolites, it is not possible to conclude or predict that endophytes may display the expected bioactivity under field conditions. Such compounds are better suited for further characterization and purification and are also more important in routine testing toolkits before commercially introducing novel endophyte associations. Furthermore, the pipeline for bioassay-guided antifungal metabolite isolation, annotation, and analysis can be used in future studies to screen for antifungal activity of other *Epichloë* sp. endophyte strains. Finally, the highly reproducible nature of metabolite biosynthesis both in in vitro culture as well as *in planta* indicates that the methodology can be replicated and developed further to isolate and purify antifungal metabolites from *Epichloë* sp. endophyte strains efficiently. In this study, compounds from refined fractions were annotated based on their unique mass, *m/z* and retention time (RT), a more accurate characterization is possible with further purification and structure analysis.

## 4. Materials and Methods

### 4.1. Plant Material

All plant material was obtained from a glasshouse maintained (natural day lengths and a mean temperature of 22 °C) collection at the Agriculture Victoria, Bundoora, Victoria, Australia. Prior to harvesting for metabolomics analysis, the presence and identity of endophyte strains was confirmed by the extraction of DNA using Qiagen MagAttract DNA (Qiagen, Hilden, Germany) and SNP-based diagnostic testing, KASP analysis (Kompetitive Allele Specific PCR) (KASP—, LGC Genomics, Teddington, UK). 

### 4.2. Fungal Cultures

#### 4.2.1. Endophyte Cultures

*Epichloë* sp. endophyte strains were isolated by Fernando et al., (2020) from pre-inoculated perennial ryegrass plants maintained at the Agriculture Victoria, Bundoora, Victoria, Australia [45]. Isolates were maintained in culture and stored as solid cultures on potato dextrose agar (PDA) (Sigma-Aldrich, Castle Hill, NSW, Australia) at 22 °C in the dark [85] and sub-cultured every two months. The identity of the endophyte strain was confirmed by KASP analysis prior to the study, as described by Fernando et al., (2020) [45]. 

#### 4.2.2. Pathogen Cultures

A culture of *Ceratobasidium* sp. (VPRI 22537), the causative phytopathogen of yellow patch disease in perennial ryegrass and sharp eyespot in *Triticum aestivum* (wheat), was obtained from the National Collection of Fungi, Bundoora Herbarium, Victoria. The pathogen was stored as a solid culture on PDA (Sigma-Aldrich, Castle Hill, NSW, Australia) at 22 °C in the dark and sub-cultured every two months to maintain stocks. The genus of the pathogen was confirmed by ITS sequence analysis as described by Fernando et al., (2020) [45].

### 4.3. Chemicals

All extraction and mobile-phase solvents were HPLC grade; methanol (99.9% pure; Fisher chemicals, Fair Lawn, NJ, USA), acetonitrile with 0.1% formic acid (≥99.9% pure; Sigma-Aldrich CHROMASOLV^®^, Castle Hill, NSW, Australia, HPLC grade), dichloromethane (DCM) (≥99.9% pure; Burdick & Jackson, HPLC grade), water with 0.1% formic acid (Fisher Chemical, Fair Lawn, NJ, USA), and ammonium formate (99.0% pure; Sigma-Aldrich, St. Louis, MO, USA). C18 powder (Grace Davidson Discovery Sciences, CA, USA) was used for the adsorption of extracts. For the positive control, a 1000 ppm solution of antifungal compound carbendazim (97% pure; sourced from Sigma-Aldrich, Castle Hill, NSW, Australia) was prepared in a 4:1 ration of (*v*/*v*) methanol: H_2_O. 

### 4.4. Extraction and Fractionation of In Vitro Culture

#### 4.4.1. Crude Extracts

Highly active crude media supernatant (MS) extracts from liquid cultures of *Epichloë* sp. endophyte strains NEA12 and NEA23, described by Fernando et al., (2020), were used for further fractionation and isolation of antifungal metabolites [45]. MS extracts collected from two-week-old NEA12 and NEA23 PDB cultures are termed NEA12 MS and NEA23 MS, respectively.

#### 4.4.2. Solid Phase Fractionation of Crude Extracts

Aqueous methanolic media supernatant extract was dried in the rotary evaporator and weighed (Table 3). Highly active crude media supernatant (MS) extracts were adsorbed to C18 (C18 Silica gel spherical, Sigma-Aldrich, St. Louis, MO, USA) in a 1:10 *v/w* ratio and dried in the rotary evaporator (Heidolph Laborota 4000, Heidolph Instruments, Schwabach, Germany) at 40 °C to obtain a fine yellow powder. The extract adsorbed to C18 was tightly packed in a 1 L preparative column for crude fractionation in the HPLC system (Dionex Ultimate 3000 (Dionex, Sunnyvale, CA, USA)). Fractionation was carried out sequentially using seven solvent systems (Table 3), where the column was flushed with one column (1 L) volume of each solvent system to obtain seven crude fractions. The obtained crude fractions (CF1–CF14) were dried in the rotary evaporator at 40 °C and weighed for dry weight. Fractions were then resuspended in appropriate solvents to give a concentration of 1 g/mL. From each crude fraction, 60 µL was aliquoted into HPLC vials separately for further analysis using LCMS.

#### 4.4.3. Agar Well Diffusion Assay for Crude Fractions

Agar well diffusion assays were conducted for crude fractions on PDA plates according to the method described by Fernando et al. 2020 [45]. A small plug of *Ceratobasidium* sp. mycelia (5 × 5 mm) was transferred onto the center of the PDA plate, and 40 µL of the extract was placed in each of the four equidistant wells (4 mm diameter). Bioassay plates were prepared in replicate (*n* = 5). Plates were incubated at 22 °C for eight days and observations were taken daily from day three. Control assays included sterile distilled water, 4:1, *v*/*v* methanol: H_2_O, DCM, respective MS (NEA12 or NEA23) extract and carbendazim (1 mg/mL) (97%) (methyl benzimidazol-2-ylcarbamate; Sigma-Aldrich, Castle Hill, NSW, Australia). The growth of the fungal pathogen *Ceratobasidium* sp., was observed daily for up to 8 days. The measurements of the pathogen growth were analyzed using ImageJ 1× 5 mm) was transferred onto the center of the PDA plate, and 40 µL of the extract was placed in each o software (NIH, Bethesda, MA, USA) [92] and expressed as area (cm^2^). A one-way-ANOVA statistical analysis was performed on day 6 using Minitab^®^ 19 Statistical Software Minitab (LLC, State College, PA, USA, a generated Tukey’s Post-Hoc test was used to separate group treatments with statistical significance, and Tukey’s simultaneous tests for differences of means at 99% confidence level was used to express the significant differences of the bioactivity among crude fractions. 

#### 4.4.4. Fractionation Using Semi Preparative High-Performance Liquid Chromatography

Moderate to strongly bioactive crude fractions (CF) were further purified by fractionation. Fractions were dried, weighed, and resuspended in 4:1, *v*/*v* methanol: H_2_O to obtain a concentration of 1 g/mL. A Dionex Ultimate 3000 solvent delivery system (Dionex, Sunnyvale, CA, USA) was equipped with a binary pump, photodiode array detector (PDA 3000), attached to a Rheodyne Model 7725 injector with a 1 mL injector loop and operated using Chromeleon version 6.8 software (Dionex, Sunnyvale, CA, USA). MiliQ water methanol/acetonitrile (Sigma-Aldrich CHROMASOLV^®^, Castle Hill, NSW, Australia, HPLC grade) was used as mobile phases A and B. Using a C18 semi prep column Thermo Scientific Hypersil gold 5 µm C18 (2) 100 Å, 150 × 10 mm column, crude fractions were separately subjected to further fractionation to obtain refined fractions. 

### 4.5. Media Supernatant Extraction of Endophyte Cultures

#### 4.5.1. Preparation of Liquid Cultures

Liquid PDB (Potato Dextrose Broth) (Sigma-Aldrich, Castle Hill, NSW, Australia) cultures were prepared by using a sterile scalpel blade to cut a small section (1 × 1 cm) of *Epichloë* sp. mycelia at the periphery and place it into a sterile 1.7 mL Eppendorf tube containing 500 µL PDB. A sterile plastic pestle was used to gently grind the mycelia and agar. Another 500 µL PDB medium was added to the Eppendorf tube containing the ground endophyte. The ground mycelia, in 15 µL aliquots, was distributed into 100 mL culture vessels containing 30 mL of PDB. A total of 28 liquid culture replicates were prepared for each endophyte strain. Culture vessels were incubated in the dark at 22 °C on a shaker at 150 rpm (Ratek OM11, Adelaide, SA, Australia) for period of 14 days.

#### 4.5.2. Extraction

Two-week-old liquid cultures were transferred to sterile 50 mL falcon tubes and centrifuged at 3836× *g* for 15 min at 4 °C (SIGMA Laborzentrifugen 4-16KS, Osterode, Germany). The media supernatant was then separated from the cell pellet, frozen, and lyophilized (Freeze dryer ALPHA, Christ, Germany). Samples were weighed to 20 mg ± 2 mg and extracted using methanol: H_2_O (*v*/*v*, 4:1, 1:3 *w/v*). For extraction, samples were vortexed (5 min) (Ratek multi-tube vortex mixer, MTV1, Boronia, VIC, Australia) and sonicated (10 min) (SoniClean, 250TD, Thebarton, SA, Australia) with the solvent. A 60 µL aliquot of the extracts were transferred into HPLC vials ready for LCMS analysis. Replicated (*n* = 28) small-scale extracts collected from two-week-old NEA12 and NEA23 PDB cultures are termed NEA12 ME and NEA23 ME, respectively. Separately, PDB media only controls were prepared; 30 mL sterile PDB media samples (*n =* 18) were freeze-dried and extracted (PDB ME). 

### 4.6. Plant Material Extraction

Pseudostems from mature plants were harvested into collection tubes and freeze-dried for 48 h before being grinded to a fine powder (28 Hz for 2 min, Geno Grinder, NJ, USA). The freeze-dried, ground plant material (20 mg ± 0.2 mg) was extracted twice with methanol: water (80:20, *v*:*v*, 1 mL). Extracts were combined, dried, and reconstituted in methanol: water (80:20, *v*:*v*, 200 µL). Replicated extracts collected from mature pseudostems of perennial ryegrass-NEA12 (*n =* 48) and perennial ryegrass-NEA23 (*n* = 30) symbiota are termed NEA12 PE and NEA23 PE, respectively.

### 4.7. LCMS Data Acquisition and Analysis

All extracts were analyzed on a Vanquish Ultra-High Performance Liquid Chromatography (UHPLC) system (Thermo Fisher Scientific, Bremen, Germany) with a binary pump, autosampler, and temperature-controlled column compartment coupled with a Thermo Fisher Q Exactive Plus mass spectrometer (QE MS) (Waltham, MA, USA; Thermo, Bremen, Germany). Extracts were separated using a Thermo Scientific Hypersil Gold 1.9 μm, 150 × 2.1 mm column (Thermo Fisher Scientific, Waltham, MA, USA). Chromatographic separation was performed by gradient elution using water with 0.1% formic acid (Sigma-Aldrich CHROMASOLV^®^, Castle Hill, NSW, Australia, Castle Hill, NSW, Australia, HPLC grade) as Solvent A and acetonitrile with 0.1% formic acid (Sigma-Aldrich CHROMASOLV^®^, Castle Hill, NSW, Australia, HPLC grade ≥ 99.9%) as Solvent B at a flow rate of 0.3 mL/min. Initial conditions were 98% A, which was then progressed to linear gradient to 100% B over 11 min, and this was maintained for 4 min before returning to the initial gradient conditions. The injection volume was 3 μL. 

The MS detector was operated in FT positive and negative mode using a full-scan with a mass range of *m/z* 80–1200. The ESI drying gas (N_2_) was set at a flow rate of 7 L/min at 350 °C, and the nebulizer gas (N_2_) pressure was set at 45 psig. Capillary, fragmentor, and skimmer voltage was set at 3500 V, 175 V, and 65 V, respectively. Prior to data acquisition, the system was calibrated with Pierce^®^ LTQ Velos ESI Positive and Negative Ion Calibration Solution (Thermo Fisher Scientific™). Mass spectrometry data were acquired using Thermo Xcalibur V. 2.1 (Thermo Fischer Scientific Inc., San Jose, CA, USA) and data was analyzed using Thermo Xcalibur Qual Browser V. 2.1. 

Acquired data were further analyzed using Refiner MS and Analyst (Genedata, Basel) [11,67]. Using Refiner MS module of Genedata Expressionist^®^ spectral data of the media, plant extracts (PE), media supernatant extracts (ME), and refined fractions (RF) were separately processed: (1) Selection of positive mode data; (2) blank subtraction by Blank Sample Intensity Factor 1 to eliminate metabolites from the blanks; (3) chemical noise subtraction by a retention time (RT) window of 151 scans at 50% quantile and different intensity threshold for each type of extracts (intensity thresholds; RF: 10,000,000; PE and ME: 5000); (4) intensity thresholding using a clipping method and an intensity threshold of 100; (5) RT structure removal by removing signals that did not extend to at least six scans; (6) chromatogram RT alignment using a pairwise alignment-based tree and a maximum RT shift of 0.1 min and; (7) saved all data files as snapshots for three extract types separately.

Metabolite annotation was carried out together for extracts: (1) loading data from saved snapshot files; (2) chromatogram RT alignment using a pairwise alignment-based tree and a maximum RT shift of 0.1 min; (3) chromatogram peak detection using a five scans summation window, a minimum peak size of 0.1 min, a maximum merge distance of 0.005 Da, a boundary merge strategy, a maximum gap/peak ratio of 7% with moving average smoothing over three scans for peak RT splitting; (4) chromatogram isotope clustering using RT of 0.02 min, *m*/*z* tolerance of 0.05 Dalton, maximum missing peaks of 1 and a first allowed gap position of 3, with a peptide isotope shaping envelope fitting method where the maximum charge was 1; (5) singleton filter for removal of peaks that do not belong to a cluster (Supplementary Figure S2). Features were exported to the Analyst module of Genedata Expressionist^®^ (Supplementary Figure S3). Using Genedata analyst all data was analyzed to create Venn diagrams and compare metabolites in extracts from endophytes grown in vitro (ME) and *in planta* (PE) with refined fractions. Features from sterile PDB extracts were filtered out from in vitro (ME) extract features to eliminate metabolites from the media. For the plant extracts of two endophyte strains, a *t*-test was used to generate Benjamini-Hochberg adjusted *p* values (Q value) to identify the significance of relative abundance of metabolites. The directed effect size was calculated by obtaining the ratio of average relative abundance of desired extracts.

## 5. Conclusions

Natural product isolation and structure elucidation can be time-consuming and labor-intensive, particularly if the bioactive metabolite is present in very small amounts. The workflow developed in this study allows for testing of endophyte bioactivity while ensuring that the metabolite is expressed *in planta* and is so useful for field deployment. This method also provides a shortlist of potential bioactives (in this case 61 metabolites out of more than 20,000 *in planta*) that can be used to explore endophyte-mediated disease resistance in populations of perennial ryegrass-endophyte symbiota. Metabolites present in vitro and absent *in planta* are also of interest as they may be induced in response to environmental stimuli. Further investigation is needed to determine the presence and abundance of these metabolites in symbiota in response to challenges by disease-causing pathogens. Purification, structural characterization, and confirmation of the bioactivity of isolated compounds will allow for the identification of metabolites associated with endophyte-mediated disease control/resistance in pasture and turf grasses. 

## Figures and Tables

**Table 1:** One-way ANOVA analysis results for pathogen growth.

Endophyte Strain	Source	DF ^1^	Seq SS ^2^	Contribution	Adj SS ^3^	Adj MS ^4^	F-Value	*p*-Value
	Factor	11	7054.1	96.63%	7054.1	641.282	122.33	<0.001
NEA12	Error	47	246.4	3.37%	246.4	5.242		
	Total	58	7300.5	100.00%				
	Factor	11	7948.0	97.47%	7948.0	722.550	168.00	<0.001
NEA23	Error	48	206.4	2.53%	206.4	4.301		
	Total	59	8154.5	100.00%				

^1^ Degrees of freedom, ^2^ Sequential sums of squares, ^3^ Adjusted sequence sum of squares and ^4^ Adjusted mean squares.

**Table 2:** Metabolites present in refined fractions extracted from NEA12 MS and NEA23 MS that are also present in the metabolomes of perennial ryegrass-endophyte symbiota (NEA12 PE and NEA23 PE).

Metabolite No.	Mass	*m*/*z*	RT	Presence/Absence in Refined Fractions		Presence *in Planta* (Number of Symbiota) ^1^		Q-Value ^2^	Directed Effect Size (NEA23 PE: NEA12 PE)
				NEA23 RFs	NEA12 RFs	NEA23 PE	NEA12 PE		
M1	242.0412	243.0485	2.91	+	−	9	23	0.189	NA
M2	255.2558	256.2631	11.19	+	−	30	47	0.004	1.59
M3	364.1280	365.1353	9.14	+	+	30	48	0.891	NA
M4	365.3625	366.3697	12.95	+	−	30	48	<0.001	1.47
M5	365.3652	366.3725	12.84	+	−	30	48	<0.001	1:3.92
M6	365.3682	366.3755	12.95	+	−	30	48	<0.001	1.57
M7	367.4173	368.4245	13.50	+	−	30	48	<0.001	1:1.68
M8	407.3153	408.3226	8.36	−	+	0	9	NA	NA
M9	426.2376	427.2449	10.80	+	−	25	40	0.191	NA
M10	426.2375	427.2448	10.90	+	−	30	45	0.749	NA
M11	452.3370	453.3442	8.61	−	+	3	4	0.316	NA
M12	452.3344	453.3417	8.70	−	+	3	3	0.661	NA
M13	452.3341	453.3414	8.72	−	+	0	2	NA	NA
M14	457.3394	458.3467	9.92	+	−	29	48	0.919	NA
M15	462.2965	463.3038	9.83	+	−	30	48	0.792	NA
M16	466.3496	467.3569	9.25	−	+	30	47	0.248	NA
M17	483.3763	484.3835	9.24	+	+	4	47	0.363	NA
M18	496.3602	497.3674	8.71	+	+	15	39	0.004	1:7.06
M19	527.4024	528.4096	9.24	+	+	20	43	<0.001	2.49
M20	532.3574	533.3647	9.24	+	+	8	48	0.772	NA
M21	546.3005	547.3077	11.85	+	−	17	9	0.024	1:2.39
M22	571.4285	572.4358	9.24	+	+	5	24	0.945	NA
M23	576.3837	577.3909	9.23	+	+	3	38	<0.001	3.93
M24	589.4180	590.4253	9.89	+	−	30	40	<0.001	5.12
M25	590.4236	591.4308	11.79	+	−	30	48	<0.001	1:2.20
M26	594.3594	595.3666	9.82	+	−	30	48	<0.001	1.31
M27	594.3744	595.3817	9.90	+	−	30	48	<0.001	1.82
M28	607.4645	608.4718	11.83	+	−	3	10	0.234	NA
M29	615.4546	616.4618	9.24	+	+	3	17	<0.001	11.86
M30	620.4100	621.4173	9.24	+	+	7	29	0.218	NA
M31	634.4644	635.4717	11.85	+	−	4	2	NA	NA
M32	651.4907	652.4980	11.85	+	−	1	12	NA	NA
M33	662.4203	663.4276	9.80	+	−	30	48	<0.001	1.71
M34	662.4171	663.4243	14.30	+	+	30	48	<0.001	1:4.53
M35	662.4447	663.4520	14.38	+	+	30	48	<0.001	1.25
M36	663.4473	664.4546	14.33	+	+	30	48	<0.001	1:1.71
M37	678.3797	679.3869	11.94	+	−	30	48	0.235	NA
M38	678.4871	679.4944	11.95	+	−	27	48	0.453	NA
M39	684.4323	685.4395	14.23	−	+	29	48	<0.001	1:9.06
M40	684.4297	685.4369	14.53	−	+	30	48	<0.001	1.58
M41	684.4313	685.4385	14.58	−	+	30	48	<0.001	1.64
M42	685.4159	686.4232	14.29	−	+	30	48	<0.001	1:3.27
M43	695.5170	696.5243	11.87	+	−	1	9	NA	NA
M44	695.5172	696.5245	11.96	+	−	5	32	0.086	NA
M45	721.4687	722.4759	11.97	+	−	24	37	0.174	NA
M46	762.5085	763.5158	14.59	−	+	30	48	0.177	NA
M47	762.5091	763.5164	14.68	−	+	30	48	<0.001	1.74
M48	762.5095	763.5168	14.74	−	+	30	47	<0.001	2.04
M49	762.5071	763.5144	14.38	+	+	30	48	<0.001	1.23
M50	762.5090	763.5163	14.54	−	+	30	48	<0.001	1.54
M51	763.4952	764.5024	14.64	−	+	30	48	<0.001	1.80
M52	763.5110	764.5182	14.42	+	+	30	48	<0.001	2.48
M53	763.5109	764.5182	14.33	+	+	30	48	<0.001	1:1.90
M54	787.5037	788.5109	12.10	+	−	26	39	<0.001	1:1.19
M55	788.5186	789.5259	11.90	+	−	30	43	0.919	NA
M56	788.5210	789.5283	11.94	+	−	29	40	0.419	NA
M57	802.5324	803.5397	12.41	+	−	30	48	0.607	NA
M58	802.5319	803.5392	12.46	+	−	30	48	<0.001	1.24
M59	802.5335	803.5408	12.26	+	−	30	48	<0.001	1:1.34
M60	802.5342	803.5415	12.33	+	−	30	48	0.960	NA
M61	858.5970	859.6043	12.59	+	−	3	6	<0.001	1.18

^1^*In planta* extracts are from pseudo stems harvested from glasshouse maintained perennial ryegrass-endophyte symbiota. NEA12 populations (*n* = 48): Bronsyn (*n* = 24) and Alto (*n* = 24); NEA23 populations (*n* = 30): Bronsyn (*n* = 15) and Trojan (*n* = 15). ^2^ Benjamini-Hochberg adjusted *p* values.

**Table 3:** Solvent systems used in fractionation of media supernatant (MS) extracts collected from *Epichloë* sp. strains NEA12 and NEA23 grown in PDB.

Crude MS Extract Strain	Crude Fraction	Water (%)	Methanol (%)	DCM (%)	Dry Weight/(g)
NEA12 MS	CF1	100	0	-	40.69
	CF2	80	20	-	10.17
	CF3	50	50	-	4.36
	CF4	20	80	-	22.01
	CF5	0	100	0	14.23
	CF6	-	50	50	7.38
	CF7	-	0	100	10.41
NEA23 MS	CF8	100	0	-	32.87
	CF9	80	20	-	8.13
	CF10	50	50	-	7.83
	CF11	20	80	-	7.26
	CF12	0	100	0	3.29
	CF13	-	50	50	2.30
	CF14	-	0	100	3.51

## Data Availability

Data is available from authors upon reasonable request.

## Supplementary Materials

The following are available online at https://www.mdpi.com/article/10.3390/metabo12010037/s1, Table S1: Metabolites present in refined fractions extracted from NEA12 MS and NEA23 MS that are also present in the metabolomes of in vitro cultures (NEA12 ME and NEA23 ME); Table S2: Metabolite distribution in refined fractions derived from bioactive crude fractions; Figure S1: Relative abundance of metabolites (a) M1-M15, (b) M16-M30, (c) M31-M45 and (d) M46-M61 present in NEA12 (NEA12_PE) and NEA23 (NEA23_PE) plant extract metabolomes and in refined bioactive fractions of NEA12 (RF1-RF7) and NEA23 (RF8-RF10). Error bars represent + standard error; Figure S2: LCMS data imported to Refiner MS and processed for data refining using suitable parameters (a) positive ion data of plant extracts, (b) processed plant extract data after blank subtraction, chromatogram noise subtraction, intensity thresholding, RT structure removal and chromatogram RT alignment, (c) workflow for data processing; Figure S3: Refined data imported from (a) plant extracts (b) media supernatant extracts (c) refined fractions to be annotated together in Refiner MS. (d) Annotated data after chromatogram RT alignment, chromatogram peak detection, chromatogram isotope clustering followed by singleton filter and (e) workflow for data processing.
