# Supplementary material for: A Metabolomic Study of Epichloë Endophytes for Screening Antifungal Metabolites
Source: Metabolites. 2022 Jan 4;12(1):37. doi: 10.3390/metabo12010037 (PMC8781816; doi:10.3390/metabo12010037)
Supplement: Supplementary file 1 [file metabolites-12-00037-s001.zip › metabolites-1506328-supplementary.pdf]

Supplementary Materials: Bioprospecting *Epichloë* sp. Endophytes for the Discovery of Antifungal Metabolites

Krishni Fernando, Priyanka Reddy, Kathryn M. Guthridge, German C. Spangenberg, Simone J. Rochfort

Supplementary Table S1: Metabolites present in refined fractions extracted from NEA12 MS and NEA23 MS that are also present in the metabolomes of *in vitro* cultures (NEA12 ME and NEA23 ME).

Name	Mass	m/z	RT	Presence/absence in refined fractions		Presence in vitro (number of cultures)		Q-Value	Directed effect size (NEA23 ME: NEA12 ME)
				NEA23 RF	NEA12 RF	NEA23	NEA12		
M1	242.0412	243.0485	2.91	+	-	0	0	NA	NA
M2	255.2558	256.2631	11.19	+	-	28	27	0.048	NA
M3	364.1280	365.1353	9.14	+	+	28	27	<0.001	1.20
M4	365.3625	366.3697	12.95	+	-	28	27	<0.001	1:1.18
M5	365.3652	366.3725	12.84	+	-	28	27	<0.001	1:9.67
M6	365.3682	366.3755	12.95	+	-	28	27	<0.001	1:1.2
M7	367.4173	368.4245	13.50	+	-	28	27	0.046	NA
M8	407.3153	408.3226	8.36	-	+	3	12	0.171	NA
M9	426.2376	427.2449	10.80	+	-	28	27	<0.001	1:1.97
M10	426.2375	427.2448	10.90	+	-	28	27	0.001	1:1.23
M11	452.3370	453.3442	8.61	-	+	28	27	0.206	NA
M12	452.3344	453.3417	8.70	-	+	28	27	<0.001	1.82
M13	452.3341	453.3414	8.72	-	+	28	27	<0.001	2.57
M14	457.3394	458.3467	9.92	+	-	28	27	0.145	NA
M15	462.2965	463.3038	9.83	+	-	28	27	<0.001	1:2.53
M16	466.3496	467.3569	9.25	-	+	22	27	0.037	NA
M17	483.3763	484.3835	9.24	+	+	28	27	<0.001	1:3.48
M18	496.3602	497.3674	8.71	+	+	0	8	NA	NA
M19	527.4024	528.4096	9.24	+	+	28	27	<0.001	1:3.53
M20	532.3574	533.3647	9.24	+	+	28	27	<0.001	1:3.56
M21	546.3005	547.3077	11.85	+	-	28	27	<0.001	1:1.10
M22	571.4285	572.4358	9.24	+	+	27	27	0.006	1:3.74
M23	576.3837	577.3909	9.23	+	+	28	27	<0.001	1:4.12
M24	589.4180	590.4253	9.89	+	-	28	27	<0.001	1:1.08
M25	590.4236	591.4308	11.79	+	-	0	2	NA	NA
M26	594.3594	595.3666	9.82	+	-	28	27	<0.001	1:1.61
M27	594.3744	595.3817	9.90	+	-	28	27	0.212	NA
M28	607.4645	608.4718	11.83	+	-	6	12	0.775	NA
M29	615.4546	616.4618	9.24	+	+	27	27	<0.001	1:4.60
M30	620.4100	621.4173	9.24	+	+	27	27	<0.001	1:4.28
M31	634.4644	635.4717	11.85	+	-	15	24	0.309	NA
M32	651.4907	652.4980	11.85	+	-	13	13	0.951	NA
M33	662.4203	663.4276	9.80	+	-	28	27	<0.001	1.07
M34	662.4171	663.4243	14.30	+	+	28	27	<0.001	1:2.30
M35	662.4447	663.4520	14.38	+	+	28	27	0.021	NA
M36	663.4473	664.4546	14.33	+	+	28	27	<0.001	1:1.92
M37	678.3797	679.3869	11.94	+	-	28	27	0.717	NA
M38	678.4871	679.4944	11.95	+	-	0	0	NA	NA

M39	684.4323	685.4395	14.23	-	+	1	10	0.659	NA
M40	684.4297	685.4369	14.53	-	+	28	27	<0.001	1.42
M41	684.4313	685.4385	14.58	-	+	28	27	<0.001	1.47
M42	685.4159	686.4232	14.29	-	+	28	27	<0.001	1:1.72
M43	695.5170	696.5243	11.87	+	-	1	2	NA	NA
M44	695.5172	696.5245	11.96	+	-	0	0	NA	NA
M45	721.4687	722.4759	11.97	+	-	25	27	<0.001	1:3.10
M46	762.5085	763.5158	14.59	-	+	28	27	0.089	NA
M47	762.5091	763.5164	14.68	-	+	28	27	0.030	NA
M48	762.5095	763.5168	14.74	-	+	28	27	0.003	1:1.13
M49	762.5071	763.5144	14.38	+	+	28	27	0.065	NA
M50	762.5090	763.5163	14.54	-	+	28	27	0.090	NA
M51	763.4952	764.5024	14.64	-	+	28	27	0.330	NA
M52	763.5110	764.5182	14.42	+	+	28	27	0.387	NA
M53	763.5109	764.5182	14.33	+	+	28	27	<0.001	1:1.93
M54	787.5037	788.5109	12.10	+	-	0	0	NA	NA
M55	788.5186	789.5259	11.90	+	-	3	0	NA	NA
M56	788.5210	789.5283	11.94	+	-	3	0	NA	NA
M57	802.5324	803.5397	12.41	+	-	28	27	<0.001	1.71
M58	802.5319	803.5392	12.46	+	-	28	27	<0.001	1.72
M59	802.5335	803.5408	12.26	+	-	28	27	<0.001	1.33
M60	802.5342	803.5415	12.33	+	-	28	27	<0.001	2.01
M61	858.5970	859.6043	12.59	+	-	24	7	<0.001	5.91
M62	338.2356	339.2429	3.69	-	+	28	27	<0.001	1:2.54
M63	361.2333	362.2406	3.88	+	+	24	27	<0.001	1:2.41
M64	510.3759	511.3832	9.25	-	+	1	27	NA	NA
M65	540.3863	541.3936	8.71	+	+	0	7	NA	NA
M66	554.4020	555.4093	9.25	-	+	0	26	NA	NA
M67	858.5974	859.6047	12.81	+	-	19	8	0.926	NA

Supplementary Table S2: Metabolite distribution in refined fractions derived from bioactive crude fractions (CF5-CF7 and CF12-CF13)

Name	Mass	m/z	RT	Presence/absence in refined fractions									
				CF5				CF6	CF7		CF12		CF13, CF14
				RF1	RF2	RF3	RF4	RF5	RF6	RF7	RF8	RF9	RF10
M1	242.0412	243.0485	2.91	-	-	-	-	-	-	-	-	-	+
M2	255.2558	256.2631	11.19	-	-	-	-	-	-	-	+	+	-
M3	364.1280	365.1353	9.14	-	-	-	+	-	-	-	+	+	-
M4	365.3625	366.3697	12.95	-	-	-	-	-	-	-	-	+	-
M5	365.3652	366.3725	12.84	-	-	-	-	-	-	-	-	+	-
M6	365.3682	366.3755	12.95	-	-	-	-	-	-	-	-	+	-
M7	367.4173	368.4245	13.50	-	-	-	-	-	-	-	+	+	-
M8	407.3153	408.3226	8.36	-	-	-	+	-	-	-	-	-	-
M9	426.2376	427.2449	10.80	-	-	-	-	-	-	-	+	+	-
M10	426.2375	427.2448	10.90	-	-	-	-	-	-	-	+	+	-
M11	452.3370	453.3442	8.61	-	-	-	+	-	-	-	-	-	-
M12	452.3344	453.3417	8.70	-	-	-	+	-	-	-	-	-	-
M13	452.3341	453.3414	8.72	-	-	-	+	-	-	-	-	-	-
M14	457.3394	458.3467	9.92	-	-	-	-	-	-	-	-	+	-

M15	462.2965	463.3038	9.83	-	-	-	-	-	-	-	-	+	+
M16	466.3496	467.3569	9.25	-	-	-	+	-	-	-	-	-	-
M17	483.3763	484.3835	9.24	-	-	-	+	-	-	-	-	-	+
M18	496.3602	497.3674	8.71	-	-	-	+	-	-	-	-	-	+
M19	527.4024	528.4096	9.24	-	-	-	+	-	-	-	-	-	+
M20	532.3574	533.3647	9.24	-	-	-	+	-	-	-	-	-	+
M21	546.3005	547.3077	11.85	-	-	-	-	-	-	-	+	-	-
M22	571.4285	572.4358	9.24	-	-	-	+	-	-	-	-	-	+
M23	576.3837	577.3909	9.23	-	-	-	+	-	-	-	-	-	+
M24	589.4180	590.4253	9.89	-	-	-	-	-	-	-	+	+	-
M25	590.4236	591.4308	11.79	-	-	-	-	-	-	-	+	-	-
M26	594.3594	595.3666	9.82	-	-	-	-	-	-	-	-	+	-
M27	594.3744	595.3817	9.90	-	-	-	-	-	-	-	-	+	-
M28	607.4645	608.4718	11.83	-	-	-	-	-	-	-	+	-	-
M29	615.4546	616.4618	9.24	-	-	-	+	-	-	-	-	-	+
M30	620.4100	621.4173	9.24	-	-	-	+	-	-	-	-	-	+
M31	634.4644	635.4717	11.85	-	-	-	-	-	-	-	+	-	-
M32	651.4907	652.4980	11.85	-	-	-	-	-	-	-	+	-	-
M33	662.4203	663.4276	9.80	-	-	-	-	-	-	-	-	+	-
M34	662.4171	663.4243	14.30	-	-	+	-	+	+	+	-	+	-
M35	662.4447	663.4520	14.38	-	-	+	-	+	+	+	-	+	-
M36	663.4473	664.4546	14.33	-	-	+	-	+	+	+	-	+	-
M37	678.3797	679.3869	11.94	-	-	-	-	-	-	-	+	-	-
M38	678.4871	679.4944	11.95	-	-	-	-	-	-	-	+	-	-
M39	684.4323	685.4395	14.23	-	-	-	-	+	+	-	-	-	-
M40	684.4297	685.4369	14.53	-	-	+	-	+	+	+	-	-	-
M41	684.4313	685.4385	14.58	-	-	-	-	-	+	+	-	-	-
M42	685.4159	686.4232	14.29	-	-	-	-	+	-	-	-	-	-
M43	695.5170	696.5243	11.87	-	-	-	-	-	-	-	+	-	-
M44	695.5172	696.5245	11.96	-	-	-	-	-	-	-	+	-	-
M45	721.4687	722.4759	11.97	-	-	-	-	-	-	-	+	-	-
M46	762.5085	763.5158	14.59	-	-	-	-	+	+	+	-	-	-
M47	762.5091	763.5164	14.68	-	-	-	-	-	-	+	-	-	-
M48	762.5095	763.5168	14.74	-	-	-	-	-	-	+	-	-	-
M49	762.5071	763.5144	14.38	-	-	+	-	+	+	+	-	+	-
M50	762.5090	763.5163	14.54	-	-	+	-	+	+	+	-	-	-
M51	763.4952	764.5024	14.64	-	-	-	-	-	-	+	-	-	-
M52	763.5110	764.5182	14.42	-	-	+	-	+	+	+	-	+	-
M53	763.5109	764.5182	14.33	-	-	+	-	+	+	+	-	+	-
M54	787.5037	788.5109	12.10	-	-	-	-	-	-	-	+	-	-
M55	788.5186	789.5259	11.90	-	-	-	-	-	-	-	+	-	-
M56	788.5210	789.5283	11.94	-	-	-	-	-	-	-	+	-	-
M57	802.5324	803.5397	12.41	-	-	-	-	-	-	-	+	+	-
M58	802.5319	803.5392	12.46	-	-	-	-	-	-	-	+	+	-
M59	802.5335	803.5408	12.26	-	-	-	-	-	-	-	+	+	-
M60	802.5342	803.5415	12.33	-	-	-	-	-	-	-	+	+	-
M61	858.5970	859.6043	12.59	-	-	-	-	-	-	-	+	+	-
M62	338.2356	339.2429	3.69	-	-	-	-	-	-	-	-	-	+
M63	361.2333	362.2406	3.88	-	-	-	-	-	-	-	+	+	-

M64	510.3759	511.3832	9.25	-	-	-	+	-	-	-	+	+	-
M65	540.3863	541.3936	8.71	-	-	-	-	-	-	-	-	+	-
M66	554.4020	555.4093	9.25	-	-	-	-	-	-	-	-	+	-
M67	858.5974	859.6047	12.81	-	-	-	-	-	-	-	-	+	-

(a)

(b)

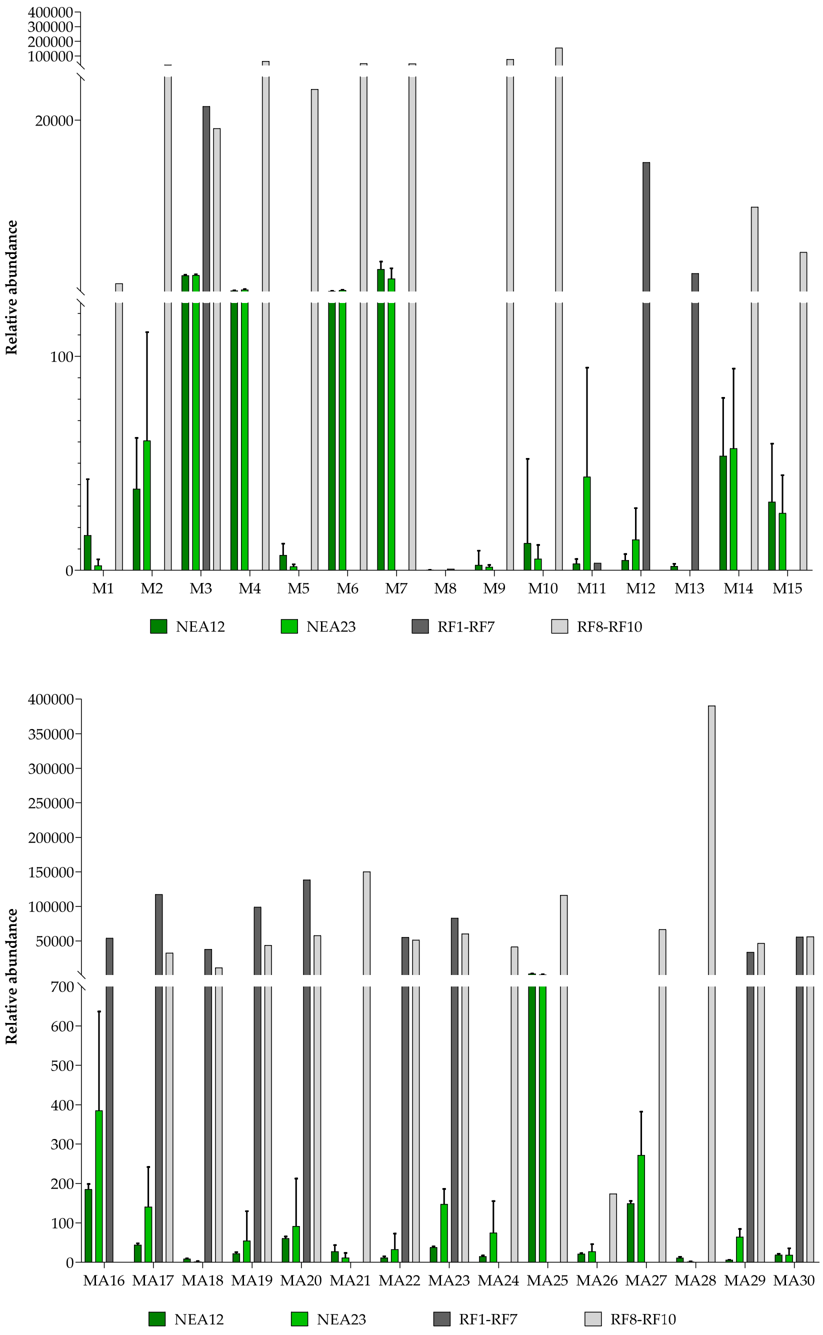

(c)

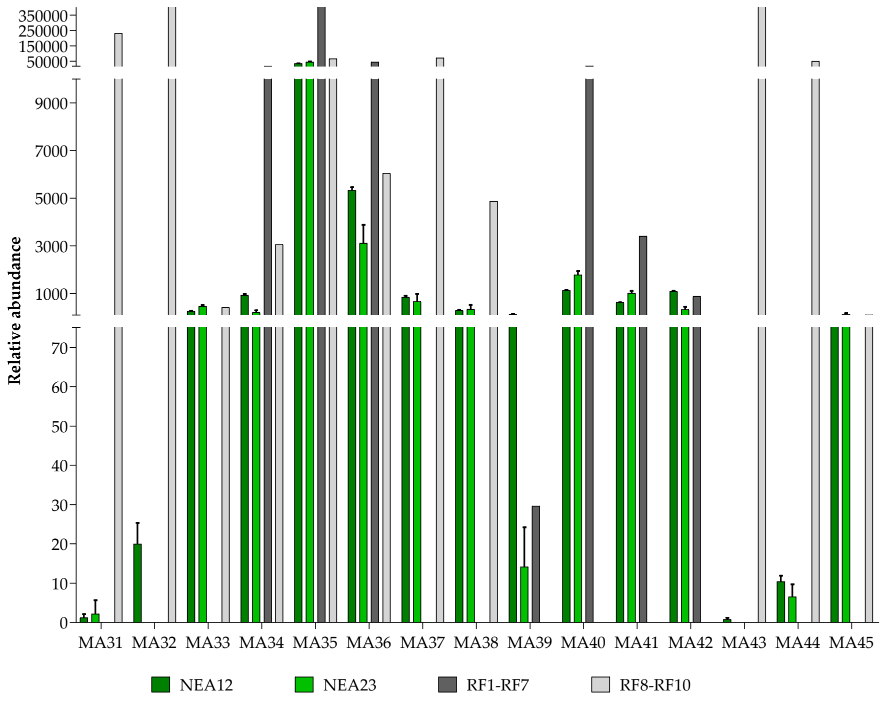

(d)

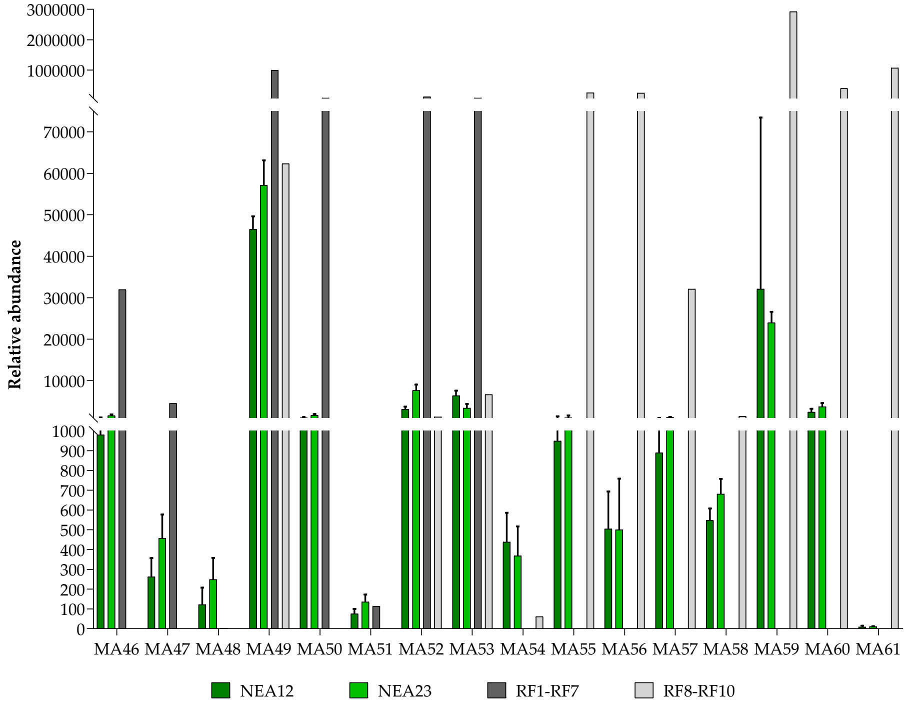

Supplementary Figure S1: Relative abundance of metabolites (a) M1-M15, (b) M16-M30, (c) M31-M45 and (d) M46-M61 present in NEA12 (NEA12 PE, n=48) and NEA23 (NEA23 PE, n=30) plant extract metabolomes and in refined bioactive fractions of NEA12 (RF1-RF7) and NEA23 (RF8-RF10). Error bars represent + standard error.

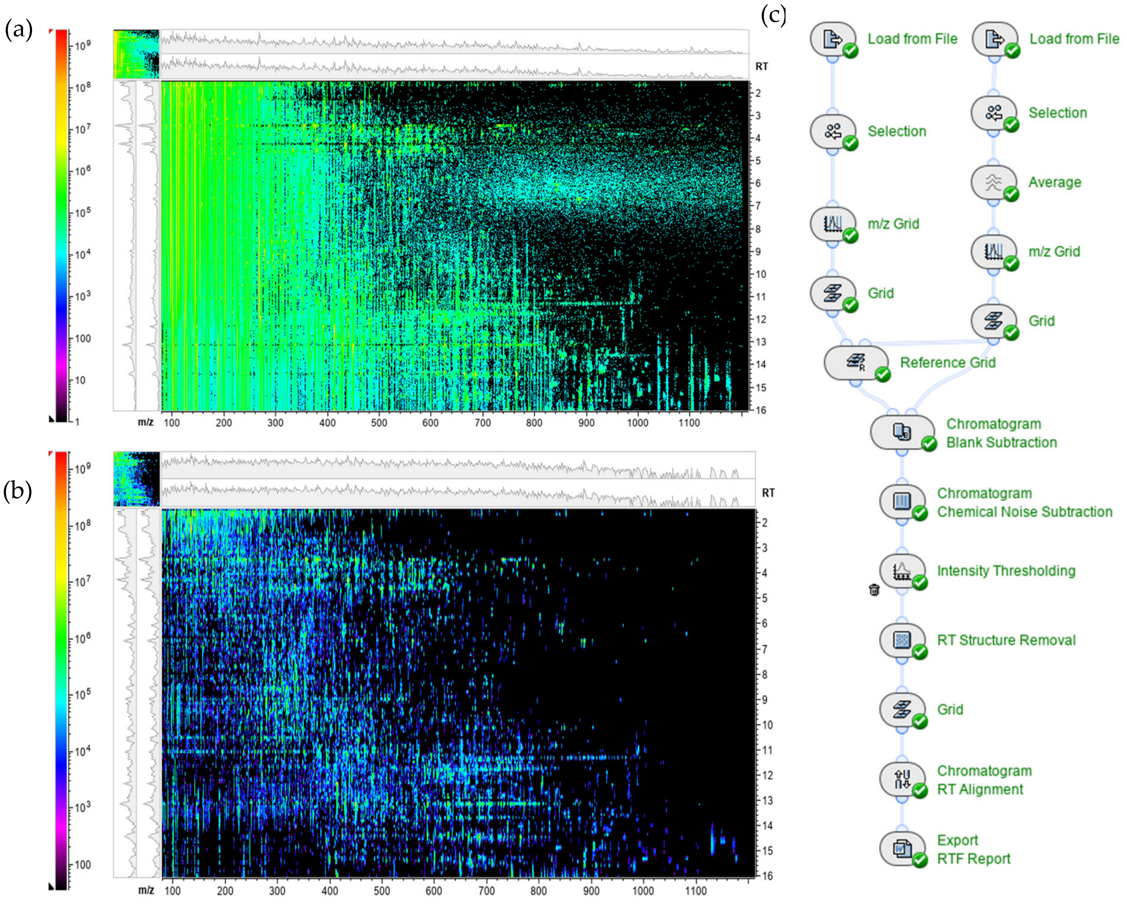

Supplementary Figure S2: LCMS data imported to Refiner MS and processed for data refining using suitable parameters (a) Positive ion data of plant extracts, (b) Processed plant extract data after blank subtraction, chromatogram noise subtraction, intensity thresholding, RT structure removal and chromatogram RT alignment, (c) Workflow for data processing

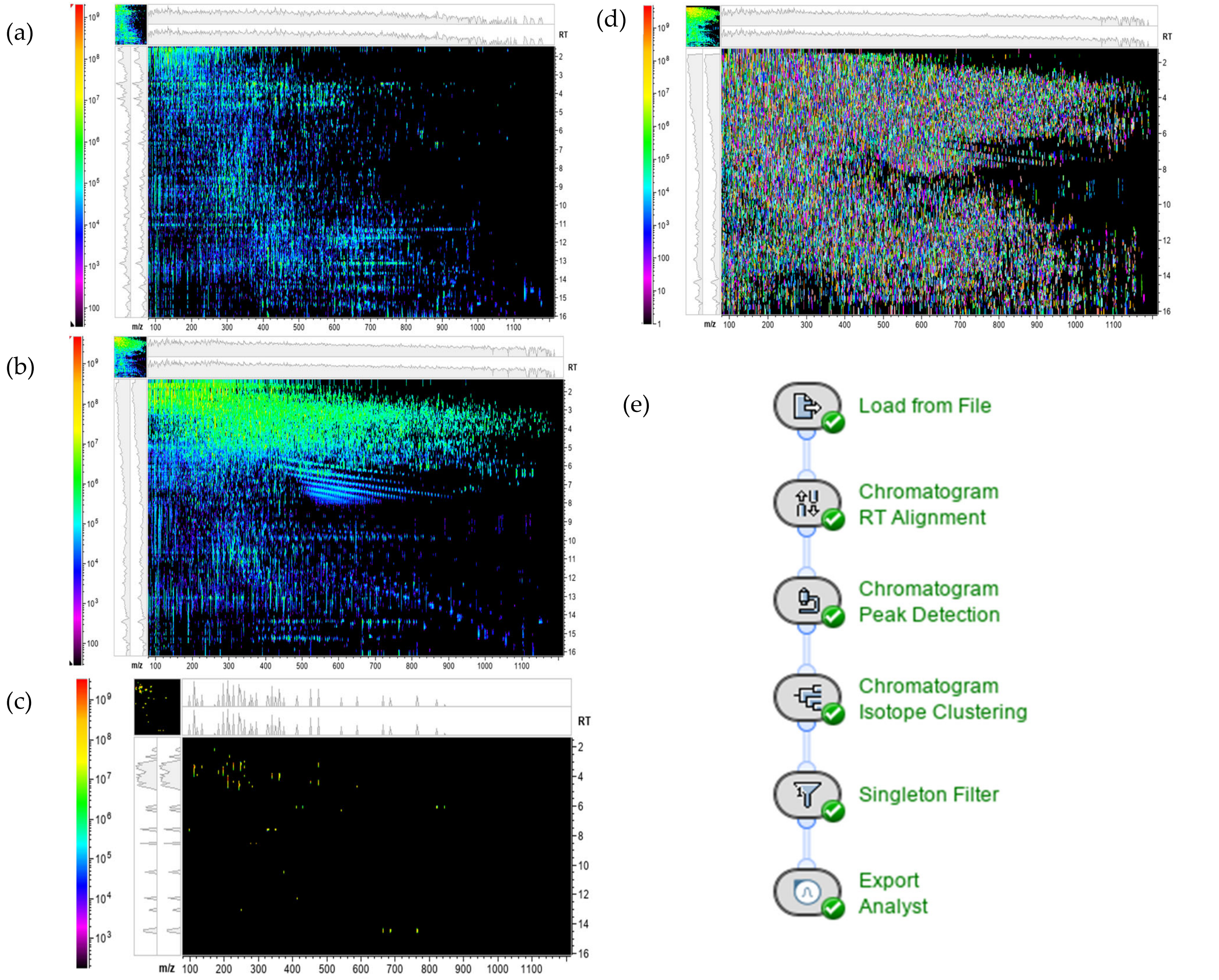

Supplementary Figure S3: Refined data imported from (a) Plant extracts, (b) Media supernatant extracts, (c) Refined fractions to be annotated together in Refiner MS, (d) Annotated data after chromatogram RT alignment, chromatogram peak detection, chromatogram isotope clustering followed by singleton filter and (e) Workflow for data processing
